# Advanced glycation end product accumulation was associated with renal function impairment in males in large health examination population

**DOI:** 10.1371/journal.pone.0351398

**Published:** 2026-06-08

**Authors:** Ryo Asaoka, Shigeki Muto, Akira Obana

**Affiliations:** 1 Department of Ophthalmology, Seirei Hamamatsu General Hospital, Hamamatsu, Shizuoka, Japan; 2 Seirei Christopher University, Hamamatsu, Shizuoka, Japan; 3 The Graduate School for the Creation of New Photonics Industries, Shizuoka, Japan; 4 Organization for Innovation and Social Collaboration, National University Corporation Shizuoka University, Hamamatsu, Shizuoka, Japan; 5 Seirei Center for Health Promotion and Preventive Medicine, Hamamatsu, Shizuoka, Japan; The University of the West Indies, JAMAICA

## Abstract

The accumulation of advanced glycation end products (AGEs) is a risk factor for renal dysfunction. However, no investigation has been conducted on the association between AGEs and renal function in health screening participants. Therefore, this study aimed to examine the association between AGEs and kidney function in health screening participants who underwent health screening examination. Overall, 1,651 health screening examinees without a history of renal dysfunction diagnosis were recruited and AGE accumulation was measured by skin autofluorescence (SAF). The association between estimated glomerular filtration rate (eGFR) and AGEs was subsequently investigated in all examinees. The mean age was 56.6 ± 9.5 years; 812 (49.1%) were males, and the mean eGFR was 73.3 ± 13.4 mL/min. Multiple regression analysis identified that AGEs were significantly negatively associated with eGFR. This finding was also observed in examinees with normal eGFR (≧ 90 mL/min/1.73 m^2^, G1 stage according to the Japanese Society of Nephrology: N = 207). In conclusion, skin AGEs were significantly negatively associated with eGFR. To clarify whether AGEs contribute to renal dysfunction progression, additional research is required.

## Introduction

Advanced glycation end products (AGEs) are the irreversible products of nonenzymatic glycation and protein and lipid oxidation [1,2]. Under normal conditions, AGEs gradually accumulate in tissues and plasma with aging [3]. However, during oxidative and/or glycemic stress, AGEs can be rapidly produced and have been associated with the progression of conditions, such as diabetes [4], atherosclerosis [5], cardiovascular diseases [6], diabetic retinopathy [7–9], age-related macular degeneration [10], and inflammatory disorders. Recently, their association with renal dysfunction has been recognized [11–15]. Smith et al. reported that increased AGE levels lead to tissue injury by activating proinflammatory and prooxidative pathways in renal dysfunction [13]. Moreover, decreased kidney function causes lower levels of AGE excretion, thereby leading to AGE accumulation [13]. Therefore, AGE accumulation induces decreased renal function, and renal dysfunction induces AGE accumulation.

To assess the concentrations of various substances within AGEs, blood tests have been commonly employed. Blood tests are costly and, owing to their invasiveness, performing them on a large scale such as in the health examination population is challenging. Nowadays, the amount of AGEs accumulated in skin can be noninvasively evaluated by measuring skin autofluorescence (SAF) [16]. Using this method, it has been reported that increased skin AGEs measured by SAF is associated with cardiovascular events and all-cause mortality in Chronic Kidney Disease (CKD) stage 3 [14,17]. Furthermore, skin AGEs measured by SAF has been reported to be an independent predictor of cardiovascular mortality in patients undergoing dialysis [11]. However, there is no report regarding the association between skin AGEs measured by SAF and renal function in health screening participants.

In this study, we aimed to examine the association between AGEs measured by SAF and kidney function in health screening participants without a history of renal dysfunction who underwent a health screening examination.

## Materials and methods

### Ethics approval

This study fully complied with the Declaration of Helsinki (64th WMA General Assembly, Fortaleza, Brazil, 2013), and the study protocols were approved by the Ethics Committee of Seirei Hamamatsu General Hospital (IRB No. 3030, 31−02). The protocols were explained in detail to all participants, who subsequently provided written informed consent to participate in the study. Informed consent was obtained from all subjects. All participant information was anonymized.

### Participants and clinical data

Participants included those who underwent health examinations at the Seirei Center for Health Promotion and Prevention Medicine from 1st September 2019 to July 2020 and consented to participate in this study. A total of 1,651 individuals with a mean age of 56.6 ± 9.5 (standard deviation, SD) years were included in this study. Those who had been diagnosed with renal dysfunction were carefully excluded. Males accounted for 812 (49.2%) of the total number of participants. The participants underwent physiological examinations, including body mass index (BMI), systolic and diastolic blood pressure (SBP and DBP), and skin AGE measurements as well as blood tests, including red blood cell (RBC) count, white blood cell (WBC) count, hemoglobin (Hb), hematocrit (Ht), platelet (Plt), albumin (Alb), estimated glomerular filtration rate (eGFR), uric acid (UA), low-density lipoprotein (LDL), high-density lipoprotein (HDL), Triglyceride (TG), hemoglobin A1c (HbA1c), and C-reactive protein (CRP) determination. eGFR was calculated using the following equation proposed by the Japanese Society of Nephrology: eGFR = 194 × Cr^−1.094^ × Age^−0.287^(×0.739 for women).

The participants’ smoking habits and alcohol consumption were also assessed. Alcohol score was counted as 1: none, 2: < 180 ml/day, 3: ≧ 180 ml/day and < 360 ml/day, 4: ≧ 360 ml/day and < 540, 5: ≧ 540 ml/day.

### Measurement of AGEs

Measurement of skin AGEs was performed using an AGE sensor (Air Water Biodesign Inc., Hyogo, Japan). The AGE sensor measures skin autofluorescence derived from AGEs and enables noninvasive assessment of skin AGE accumulation. The validity of this measurement has been supported by comparisons with skin biopsy results [16]. The measurement values are expressed in arbitrary units. The interrater reliability and two-timepoint agreement of the AGE sensor have been reported in detail, demonstrating high reliability [18]. A light source emitting light at a 365-nm wavelength excites fluorescent moieties in compounds in the skin to produce fluorescence at 440 nm. The output represents the ratio between autofluorescence in the 440-nm range and excitation light in the 365-nm range and is reported in arbitrary units (AU). Before measurement, calibration was performed according to the manufacturer’s instructions. Skin AGE measurements were performed on the palmar side of the middle finger tip.

### Statistical analysis

All results were expressed as means ± SDs. Comparisons were made using the nonpaired t-test and chi-square test for numerical and categorical variables, respectively. The association between eGFR and the values of skin AGEs, age, sex, BMI, SBP, DBP, Alb, LDL-Cho, HDL-Cho, TG, HbA1c, WBC, RBC, Hb, Ht, Plt, and CRP levels, presence of smoking habit and alcohol score, usage of antihypertensive medicine and lipid/cholesterol-lowering agents, as well as the interaction term between skin AGE and sex was analyzed using multivariate linear regression. Then, model selection was performed through a two-stage model selection. First, most important 10 parameters were selected from all 22 clinical parameters using the least absolute shrinkage and selection operator regression [19,20]. Then the final optimum model was selected using the second-order bias-corrected Akaike information criterion (AICc) index; the optimal model for the occurrence of hypotony complications was identified from all 2^10^ patterns using the 10 candidate variables. This two-stage method was necessary to reduce the total number of variables in the exhaustive AICc model selection. The AIC is a well-known statistical measurement used in model selection, whereas the AICc is a corrected version of the AIC, which provides an accurate estimation even when the sample size is small [21].

These analyses were iterated in examinees in the G1 (normal: eGFR ≧ 90 mL/min/1.73 m^2^) stage, according to the Japanese Society of Nephrology guidelines (https://jsn.or.jp/en/guideline/guideline.php)[22].

## Results

The subject data of participants including skin AGE score (measurement values obtained using AGE sensor), age, BMI, SBP, DBP, Alb, eGFR, LDL-Cho, HDL-Cho, TG, HbA1c, WBC, RBC, Hb, Ht, Plt, and CRP levels, the presence of smoking habit and alcohol score, as well as the usage of antihypertensive medicine and lipid/cholesterol-lowering agents are shown in Table 1. 340 among the 1,651 participants were patients with diabetes (Table 1).

**Table 1:** Clinical characteristics of the health screening examinees.

	Total (n = 1,651)	G1 (n = 207)
AGE score (AU)	0.49 (± 0.086)	0.48 (± 0.079)
Age (years)	56.6 (± 9.5)	50.6 (± 10.7)
BMI (kg/m^2^)	22.6 (± 3.5)	22.8 (± 4.2)
SBP (mmHg)	114.3 (± 14.0)	111.8 (± 13.0)
DBP (mmHg)	70.1 (± 9.6)	67.9 (± 8.7)
Alb (mg/dL)	4.3 (± 0.23)	4.3 (± 0.25)
UA (mg/dL)	5.3 (± 1.2)	**4.7** (± 1.1)
eGFR (mL/min/1.73 m^2^)	73.3 (± 13.4)	97.4 (± 8.0)
LDL-C (mg/dL)	126.7 (± 29.4)	121.3 (± 29.3)
HDL-C (mg/dL)	69.8 (± 17.7)	69.6 (± 17.8)
TG (mg/dL)	95.2 (± 55.1)	93.7 (± 66.1)
HbA1c (%)	5.7 (± 0.51)	5.7 (± 0.66)
WBC (/μl)	4967.3 (± 1231.2)	5117.5 (± 1340.4)
RBC (x 10^4^ /μl)	456.5 (± 41.8)	450.8 (± 41.9)
Hb (mg/dL)	13.9 (± 1.3)	13.6 (± 1.4)
Ht (%)	41.4 (± 3.5)	40.4 (± 3.5)
Plt (× 10^4^/µL)	23.7 (± 5.3)	24.8 (± 5.2)
CRP (mg/dL)	0.083 (± 0.22)	0.11 (± 0.24)
Smoking (n, %)	52 (3.1%)	15 (7.2%)
Alcohol score	1.00 (± 1.17)	0.90 (± 1.2)
antihypertensive medicine (yes)	301 (19.2%)	23 (11.1%)
lipid/cholesterol-lowering (yes)	293 (17.7%)	32 (1.5.5%)

G1: normal stage according to the Japanese Society of Nephrology guidelines, AGE; advanced glycation end products; AU, arbitrary units; BMI, body mass index; SBP, systolic blood pressure; DBP, diastolic blood pressure; Alb, albumin; UA, uric acid, eGFR, estimated glomerular filtration rate; LDL-Cho, low-density lipoprotein cholesterol; HDL-Cho, high-density lipoprotein cholesterol; TG, triglyceride; HbA1c, hemoglobin A1c; WBC, white blood cell count; RBC; red blood cell count; Hb, hemoglobin; Ht, hematocrit; Plt, platelet; CRP, C-reactive protein; Smoking, the presence of smoking habit.

The two-stage model selection with LASSO regression and AICc model selection resulted in the variables of skin AGEs, age, BMI, HbA1c, Ht, Plt, UA, CRP, smoking habits, and usage of antihypertensive medicine. Conducting the multiple regression analysis revealed a significant correlation between eGFR and AGE score, age, BMI, UA, HbA1c, Plt, and, as well as the presence of smoking habit (Table 2).

**Table 2:** Association between eGFR and 13 clinical indexes in all health screening examinees.

	Coefficient	SE	p value
**AGE score (AU)**	**−7.0**	**3.4**	**0.039**
**Age (years)**	**−0.46**	**0.034**	**<0.001**
**BMI (kg/m**^**2**^)	**0.28**	**0.092**	**0.0023**
**UA (mg/dL)**	**−3.1**	**0.27**	**<0.001**
**HbA1c (%)**	**1.64**	**0.61**	**0.0076**
**Ht (%)**	**−0.16**	**0.090**	**0.076**
**Plt (× 10** ^**4**^ **/µL)**	**0.13**	**0.057**	**0.026**
CRP (mg/dL)	2.35	1.36	0.83
**Smoking (for yes)**	**4.91**	**1.68**	**0.0035**

Other 4 clinic indexes were not selected with the LASSO regression. Bold value indicates statistical significance at *p* < 0.05. eGFR, estimated glomerular filtration rate, SE, standard error; AGE, advanced glycation end product; AU, arbitrary units; BMI, body mass index;; UA, uric acid; HbA1c, hemoglobin A1c; Ht, hematocrit; Plt, platelet; CRP, C-reactive protein; Smoking, the presence of smoking habit.

In the subanalysis of the screening examinees in the G1 stage (N = 207), AGE score, BMI and Hb were significantly associated with eGFR (Table 3).

**Table 3:** Association between eGFR and 13 clinical indexes in G1 stage.

	Coefficient	SE	p value
**AGE score (AU)**	**−14.5**	**7.0**	**0.041**
**BMI (kg/m**^**2**^)	**0.39**	**0.13**	**0.0035**
**Hb (mg/dL)**	**−1.28**	**0.40**	**0.0018**

Other 10 clinic indexes were not selected with the LASSO regression. Bold value indicates statistical significance at *p* < 0.05. eGFR, estimated glomerular filtration rate, SE, standard error; AGEs, advanced glycation end products; AU, arbitrary units; BMI, body mass index; Hb, hemoglobin.

## Discussion

In the present study, the variable significantly associated with eGFR was identified in 1,651 health screening participants without a history of renal dysfunction who underwent health screening examination. AGEs showed a significantly negative correlation with eGFR in males but not in females.

Several studies have suggested an association between AGEs and kidney dysfunction. For instance, in kidney injury, endothelial cells in the kidney stimulated by AGEs release proinflammatory mediators and adhesion molecules [23–25], which lead to tissue damage and dysfunction in the kidney. Consistent with this finding, Schutte et al. reported that elevated AGE levels were associated with lower baseline eGFR in patients with peripheral artery disease [26]. Moreover, Osawa et al. reported that AGEs is an independent predictor for diabetic nephropathy in Japanese patients with type 2 diabetes mellitus [27]. A previous study has suggested that AGEs is an independent risk factor for cardiovascular events and all-cause mortality in patients with CKD stage 3 [17]. Moreover, the current results suggest that elevated AGEs was associated with eGFR deterioration even in health screening participants (Table 1), and even in those in G1 stage (Table 2).

There have been contradicting results regarding the sex difference in AGEs [28–31]. A complex relationship exists between gender and kidney diseases, with males and females having different biological susceptibilities to the disease [32]. Previous studies have shown that although more females have renal dysfunction than males, there is a greater likelihood of males to develop kidney failure faster than females. For this reason, the male gender is a risk factor for developing kidney failure with greater severity [33,34]. The reason for these gender differences is not clearly understood; however, the current results suggested that the effect of skin AGEs on eGFR did not differ between males and females (Tables 2 and 3).

HbA1c is a product of glycation similar to AGEs, and elevated HbA1c levels were significantly associated with increased eGFR in all examinees in the current study. This may be due to the glomerular hyperfiltration in the early stage of diabetes [35]. Higher BMI was associated with increased eGFR in all examinees and also in those in G1 stage. This may be due to the obesity-related glomerulopathy [36]. Higher UA and smoking habits are established risk factor for renal dysfunction. Both of these were confirmed to have significant association with eGFR in all examinees, in the current study.

Age was significantly associated with eGFR in all examinees. A careful consideration is needed when interpreting the effect of age on eGFR, because age is a component of the eGFR formula used to estimate creatinine production and muscle mass, however inclusion as an independent covariate in the regression model is essential to adjust for biological aging, vascular integrity, and the cumulative impact of lifestyle factors—factors that influence clinical outcomes independently of renal function.

Our study had some limitations. First, this was a cross-sectional study; therefore, it was neither controlled nor randomized. Second, no detailed dietary questionnaire was used in this study. Finally, the relatively older age of the population could limit the generalizability of our results to younger populations. In addition, proteinuria, albuminuria and dietary intake of AGEs were not measured in all participants. Moreover, the very low prevalence of smoking, especially in female. A further study is needed to investigate the effect of smoking using another dataset with higher prevalence. Finally, in people> 50 years old, eGFR decline is a natural consequence of age-related glomerulosclerosis. This is certainly associated with AGEs accumulation. However, our study had several strengths, including the large number of examinees and comprehensive assessments of their clinical characteristics and laboratory data.

In conclusion, the current study suggested that AGEs were significantly negatively associated with eGFR in health screening participants. Similar result was also observed in those with normal eGFR. To clarify whether AGEs contribute to the longitudinal progression of renal dysfunction, additional research is required.
